# Determinants of quality of life among elderly patients with type 2 diabetes in northwest of iran: based on problem areas in diabetes

**DOI:** 10.3389/fendo.2022.924451

**Published:** 2022-07-22

**Authors:** Hamed Rezakhani Moghaddam, Eslam Sobhi, Aghil Habibi Soola

**Affiliations:** ^1^ Department of Public Health, Khalkhal University of Medical Sciences, Khalkhal, Iran; ^2^ Students Research Committee School of Nursing and Midwifery, Ardabil University of Medical Sciences, Ardabil, Iran; ^3^ Department of Nursing, School of Nursing and Midwifery, Ardabil University of Medical Sciences, Ardabil, Iran

**Keywords:** quality of life, elderly, psychological distress, diabetes mellitus, self-management

## Abstract

**Background:**

Diabetes is a metabolic disease characterized by chronic hyperglycemia, leading to damage to various organs of the patients and a reduction of their life expectancy and quality of life (QOL). The aim of this study was to explore the determinants of the QOL based on the Problem Areas in Diabetes (PAID).

**Methods:**

This cross-sectional study was carried out in an Iranian diabetic clinic in Ardabil. The PAID, the short form health survey (SF-12), and the sociodemographic questionnaire were all employed. Using the census sample method, 266 elderly people with type 2 diabetes from the lone diabetic clinic at Ardabil took part in this study. One-way ANOVA, t-test, one-sample Kolmogorov–Smirnov test, and multiple regression were used to analyze the data.

**Results:**

Data analysis showed that there was a statistically negative significant relationship between the QOL dimensions and the triple domains of PAID (p < 0.01). In the final model of the predictors of the QOL, treatment barriers, psychological distress related to diabetes management, the type of treatment, age, and the duration of diabetes were statistically significant predictors of the QOL dimensions (p>0.05).

**Conclusion:**

Individual characteristics and factors connected to health services should be prioritized in any intervention program aimed at improving the QOL of elderly patients with diabetes. Psychological distress should be considered in addition to regular physician visits.

## Introduction

Nowadays, factors like increasing life expectancy and declining fertility rates have led to an increase in the number of elderly population worldwide in a way that the aging of the population of the world has been introduced as one of the major public health challenges in recent years (1). Entering the elderhood, the probability of developing chronic diseases is increased significantly. Recent studies show that approximately 8% of the elderly have at least one chronic disease that makes them at risk for disability and death (2). Nearly 40% of the elderly living in the community also experience some kinds of limitations due to chronic diseases like diabetes (3, 4). One of the most debilitating diseases common among elderly is diabetes, which imposes enormous costs to the governments throughout the world (5).

Diabetes is a global health concern that affects approximately 382 million people throughout the world, and it is estimated to affect up to 592 million people by 2035 (6). According to the latest statistics reported by the World Health Organization (WHO), approximately 8 million Iranians (10.3%) have diabetes (7). The research of diabetes-related factors in Iranian elderly persons is crucial since diabetes affects approximately 22% of Iran’s elderly population (8). Many factors such as diet, blood glucose monitoring, medications, and physical activity may help patients to achieve the optimal blood sugar control (9). Diabetes is a metabolic disease characterized by chronic hyperglycemia, leading to damage to various organs of the patients and and a reduction of their life expectancy and QOL (10, 11).

The WHO has provided the comprehensive definition for QOL: “individuals’ perception of their position in life in the context of the culture and value systems in which they live and in relation to their goals, expectations, standards and concerns” (The WHOQOL Group, 1995). As defined by the WHO, it is affected in a complex way by one’s “physical and psychological health, level of independence, social relationships as well as his relationship to salient features of the environment’’ (12–14).

Various studies have identified various characteristics of the QOL. Physical, psychological, individual self-efficacy, and spiritual aspects and social participation are among these dimensions (15). Diabetes, in general, produces poor physical, social, and psychological health, which leads to restrictions in physical functioning and mental health, lowering the QOL among the elderly (16, 17). Several studies have shown that the QOL in “elderly people with diabetes” decreases compared to the non-diabetics (18, 19). Because of the difficulty of dietary restriction and the requirement to maintain self-management behavior, the QOL of patients with diabetes is significantly impacted (20). As the world’s diabetic and aging populations have grown in recent years, the need for health promotion planning to improve all aspects of life (physical, mental, social, and so on) in this group of individuals has become apparent (21, 22).

Psychological health has already been found to be effective in predicting general wellbeing, such as physical health and the QOL. The study by Nedeljkovic et al. found that the psychological status can be used to guide therapies for improving the QOL in elderly patients with diabetes, as well as strategies for maintaining health-promoting behaviors (23). Depression has been highlighted as a key component in lowering the QOL of patients with diabetes in a study by Gomez et al. (24).

Diabetic-related stress evaluation and discussion can be a useful treatment technique for addressing barriers to these patients’ therapeutic goals (25). One of the health domains of patients with diabetes affected by the disease is their psychological health, which, if neglected, may result in poor self-care behaviors (26). In addition, several studies have shown that poor glycemic control is associated with many problems such as depression and anxiety (27, 28).

Despite these studies, it has been shown that many aspects of the elderly’s personality and psychological challenges remain unknown, and many of their psychological and physical problems remain unaddressed, despite improvements in medical science and the establishment of specialized trends in geriatrics (29). Problem areas in diabetes on predicting the QOL among elderly patients with diabetes, especially in developing countries, have not been well surveyed.

This study was conducted to examine the determinants of the QOL among elderly patients with diabetes based on problem areas in diabetes. Identifying the QOL influential factors in such studies may be useful in designing interventional programs aiming at improving the QOL according to problem areas in diabetes. Therefore, our objectives in the current study were as follows: (a) to determine the predictors of QOL based on the problem areas in diabetes and (b) to measure the QOL of the elderly patients with diabetes.

## Method

### Design and sample

From July to December 2021, a cross-sectional study was conducted on a census sample of 266 elderly patients with type 2 diabetes in the only diabetes clinic in Ardabil, a mountainous city in northwestern Iran. During the study, a total of 312 “elderly patients with diabetes” were referred to the clinic. According to the inclusion criteria and their willingness to participate in the study, 266 patients participated in the study. Data were collected in a private room in the clinic. The respondents were explained about the purpose of the study, and all of them signed an informed consent form. Those subjects who gave consent to participate in the study were interviewed to complete the questionnaires. The participants were included if they were 60 years of age or older, diagnosed with type 2 diabetes by a specialist, with no cognitive and perception impairments, with no chronic diseases affecting the QOL (severe heart disease, stroke, severe neurological disorders, and end-stage renal disease), and required drug treatment due to their diabetes.

### Measures

The Problem Areas in Diabetes questionnaire (PAID) is a standard questionnaire with 20 items that measures the negative emotions related to diabetes (e.g., fear, anger, and frustration) commonly experienced by patients with diabetes. The answer to the items is based on a five-point Likert-type scale ranged from 0 to 4 (0 = not a problem, 1= minor problem, 2= moderate problem, 3= somewhat-serious problem, 4 = serious problem). Scores ranged from 0 to 80; a higher score indicates more perceived problems. The original questionnaire assesses four domains of problem areas in diabetes: 1.emotional distress, 2. treatment barriers, 3. problems related to food, and 4. the lack of social support. In different studies conducted in several countries, the number of subcategories has been changed (30, 31). The Iranian version of PAID was validated by Arzaghi et al. (32), which resulted in three domains: “psychological distress in relation to diabetes management,” “depression-related problems,” and “treatment barriers”. The internal consistency and test–retest reliability of the Iranian version of PAID was high (Cronbach’s alpha 0.94 and 0.88, respectively). In the present study, this version of PAID was used. The examples of questions were as follows: “Feeling scared when you think about living with diabetes?” (depression-related problems), “Feeling burned out by the constant effort needed to manage diabetes?” (psychological distress related to diabetes management), and “Feeling unsatisfied with your diabetes physician?” (Treatment barriers).

The Persian version of Short Form Health Survey (SF-12) was used to measure the QOL among the participants in the present study. The SF-12 includes 12 items grouped into 2 scales and 8 subscales: the physical component summary (PCS) scale includes 4 subscales (general health, physical functioning, physical role limitation, and bodily pain), and the mental component summary (MCS) scale also includes 4 subscales (mental role limitation, vitality, social functioning, and mental health). The scores in each area are scored to be in a range from 0 to 100. A higher score shows better QOL. The validity and reliability of this instrument was also confirmed in a previous study in Iran (33).

The demographic characteristics included age, gender, educational status (illiterate/literate), marital status (married/single), living status (living alone/living with a partner/living with family members), economic status (economically dependent to others/economically independent), the duration of diabetes, and the type of treatment (oral treatment/insulin therapy).

### Statistical analysis

In order to summarize and organize the data, the measures of central tendency and variability were used. The normality of data distribution was tested by the one-sample Kolmogorov–Smirnov test. The differences in the psychological wellbeing construct by demographic variables were analyzed using one-way ANOVA or the t-test. Multiple regression was performed to investigate the relationships between the sociodemographic variables and problem areas in diabetes and the QOL as the dependent variable to find a set of the best predictors. All the scores of the QOL are standardized in a range of 0 to 100 so that the worst possible score is 0 and the best possible score is 100. The correlation of study variables (scales of SF-12 and PAID domains) was tested using Pearson’s correlation coefficient test. The level of significance was considered to be 0.05 at prior. The Statistical Package for Social Sciences (SPSS) v. 22 for Windows (SPSS Inc., Chicago, IL, USA) was used to conduct all statistical analyses.

### Ethical considerations

Written informed consent was obtained from all the respondents prior to data collection. This research was approved by the Medical Ethics Committee of Ardabil University of Medical Sciences (approval number: IR.ARUMS.REC.1399.097). The study adhered to the tenets of the Declaration of Helsinki.

## Result

The information on 266 elderly patients with type 2 diabetes was included in data analysis. More than half of the respondents (57.5%) were women, and 67.3% were married. The mean age of participants was 69.48 (SD = 7.4) years with the range of 60–90 years. In addition, the mean duration of diabetes was 10.6 ± 6.3 years and the number of chronic diseases was 0.67 ± 0.47. Less than half of the elderly were living their own house with their wife (45.9%), and 65.4% had an economically independent income. More than half (64.6%) of them were illiterate, and 65.4% received oral treatment. As shown in **Table 1**, statistically significant differences were found in the QOL of the patients by age, gender, the duration of diabetes, marital status, educational status, economic status, living status, and the type of treatment (p<0.05).

**Table 1:** Demographic characteristics of elderly patients with type 2diabetes (n=266).

	Variables	Mean ± SD	N (%)	P-value
**Age**		69.48 ± 7.4		r=-0.289 p=**0.000**
**Gender**	Male	54.9 ± 25.3	113(42.5)	t=3.62 p=**0.000**
	Female	44.2 ± 22.5	153 (57.5)	
**Duration of diabetes (yrs.)**		10.6 ± 6.3		r=-0.183 p=**0.003**
**Number of Chronic Diseases**		0.67 ± 0.47		r=-0.09 p=0.13
**Marital Status**	Married	51.7 ± 24	179 (67.3)	t=2.94 p=**0.004**
	Widowed and divorced	42.5 ± 23.7	87 (32.7)	
**Educational Status**	Illiterate	43.72 ± 23.3	172 (64.6)	t=4.75 p=**0.000**
	Literate	57.98 ± 23.4	94 (35.4)	
**Economic status**	Independent	52.6 ± 24.7	174 (65.4)	t=3.67 p=**0.000**
	Dependent	41.4 ± 21.7	92 (34.6)	
**Living status**	Alone	45.7 ± 21.4	27 (10.0)	F=3.42 p=**0.034**
	With wife	52.9 ± 24.2	122 (45.9)	
	With family	45 ± 24.3	117 (44.1)	
**Kind of treatment**	Oral treatment	52.2 ± 24.1	174 (65.4)	F=5.34 p=**0.005**
	Insulin therapy	42.6 ± 23.6	75 (28.1)	
	Oral and insulin therapy	40.1 ± 22.1	17 (6.5)	

The bold values related to significant relationship (p<0.01).

The participants’ “quality of life” score averaged 48.76 ± 24.30 (min: 1.67—max: 90.83 and range: 0–100). The average “PAID” score was 32.12 ± 11.93 (min: 2—max: 60 and range: 0–80) (**Table 2**).

**Table 2:** Descriptive statistics of study variables in elderly patients with type 2 diabetes (n = 266).

Variable	Mean ± SD	Min	Max	Range
**Quality of life**	**48.76 ± 24.30**	**1.67**	**90.83**	**0–100**
PCS^1^	41.32 ± 31.24	0.00	95.83	0**–**100
MCS^2^	56.65 ± 20.84	3.33	90.00	0–100
**PAID^3^**	**32.12 ± 11.93**	**2.00**	**60.00**	**0–80**
Psychological distress related to diabetes management	10.47 ± 3.39	2.00	17.00	0–20
Depression-related problems	10.95 ± 4.56	2.00	22.00	0–24
Treatment barriers	11.97 ± 5.29	2.00	26.00	0–36

^1^Physical component summary.

^2^Mental component summary.

^3^Problem Areas in Diabetes.

Bold values means related to the total values of study variables.

According to the results, there was a negative relationship between the physical component summary (PCS) of the patients and the triple domains of PAID (p < 0.01). Statistical analysis also showed that there was a negative relationship between the mental component summary (MCS) of the patients and the triple domains of PAID (p < 0.001) (**Table 3**).

**Table 3:** Correlation of study variables in elderly patients with type 2 diabetes (n = 266).

Variable	Quality of life			
PAID	PCS		MCS	
	r	p-value	r	p-value
Psychological distress related to diabetes management	-0.195	**0.001**	-0.293	**0.000**
Depression-related problems	-0.174	**0.004**	-0.261	**0.000**
Treatment barriers	-0.258	**0.000**	-0.297	**0.000**

The bold values related to significant relationship (p<0.01).

The results obtained from the multiple regression model to predict the PCS and MCS of QOL based on problem areas in diabetes and the demographic variables are shown in **Table 4**. The results showed that the variables of age (β=0.31, p<0.001), the duration of diabetes (β=-0.12, p=0.026), the type of treatment (β=0.12, p=0.023), psychological distress (β=-0.13, p=0.031), and treatment barriers (β=-0.16, p=0.014) were significant predictors of the PCS of QOL, and age (β=0.22, p<0.001), the type of treatment (β=0.11, p=0.044), psychological distress (β=-0.22, p=0.001), and treatment barriers (β=-0.22, p=0.001) were signifcant predictors of the MCS of QOL among elderly patients with type 2 diabetes (**Table 4**).

**Table 4:** Linear regression model of the factors associated with quality- of-life (QOL) domains in elderly patients with type 2 diabetes.

Quality of life among elderly patients with type 2 diabetes								
Variable	PCS (Physical Component Summary)				MCS (Mental Component Summary)			
	SE	Beta	t	Sig	SE	Beta	t	Sig
**Age**	0.24	0.31	5.31	**0.000**	0.17	0.22	3.65	**0.000**
**Gender**	4.46	0.13	1.92	0.055	3.16	0.08	1.13	0.259
**Duration of diabetes**	0.26	-0.12	2.23	**0.026**	0.18	-0.07	1.29	0.196
**Number of chronic diseases**	3.69	0.05	0.97	0.332	2.62	0.10	1.76	0.080
**Marital status**	4.05	0.02	0.37	0.711	2.87	0.05	0.85	0.393
**Education status**	4.47	0.96	1.39	0.164	3.17	0.003	0.04	0.965
**Economic status**	4.09	-0.05	0.94	0.346	2.90	0.05	0.05	0.417
**Living status**	2.63	0.07	1.34	0.179	1.86	0.07	1.25	0.212
**Kind of treatment**	2.78	0.12	2.28	**0.023**	1.97	0.11	2.02	**0.044**
**Psychological distress**	0.56	-0.13	2.16	**0.031**	0.40	-0.22	-3.50	**0.001**
**Depression-related problems**	0.31	-0.04	0.68	0.492	0.32	-0.08	-1.16	0.244
**Treatment barriers**	0.39	-0.16	2.48	**0.014**	0.27	-0.22	-3.25	**0.001**

The bold values related to significant relationship (p<0.01).

## Discussion

The present study revealed that treatment barriers, psychological distress related to diabetes management, the type of treatment, and age were statistically significant predictors of QOL dimensions.

The age of the elderly was one of the most important determinants of the QOL in this study. This finding is in line with the results of the majority of similar studies in this field. In the investigations of Jing et al. (34), age had a significant relationship with the dimensions of the QOL; thus, as people aged older, their QOL decreased. Given that aging affects all major physiological systems, including anatomical and functional systems, and reduces scores in all elements of the elderly’s QOL, this seems to be an obvious finding. Senez et al. (35) and Mokhtari et al. (36) investigations support these findings, demonstrating a significant inverse relationship between the average QOL in all domains and the number of comorbidities.

The findings of this study showed that the domains of problem areas in diabetes are favorable predictors of the QOL, with the exception of depression-related problems. Psychological distress related to diabetes management is very common, according to studies conducted in thirteen countries, and has a major impact on diabetes patients’ QOL (37). Findings on the investigations of Eriksson (38) and Khalil Karami (39) also indicated that psychological distress is associated with poor QOL in patients with diabetes. To explain these findings, it may be claimed that when the elderly suffers from diseases like diabetes that are accompanied by psychological issues, they experience worry in the face of the sickness and a sense of powerlessness in personal and social relationships, lowering their QOL.

According to the current study, patients with highly perceived treatment barriers had a lower chance of having a good QOL. The results of various studies show that those with highly perceived obstacles have a higher risk of poor QOL (40, 41), which is consistent with the findings of this study. Diabetic caregivers should seek psychological and family counseling to help them overcome the obstacles and challenges of diabetes care as treatment barriers signal problems with regular care plans and access to doctors.

The physical dimension of patients’ QOL declined with increasing diabetes duration in the current study, and this finding has been confirmed in other studies (42, 43). Patients who have been sick for a longer period of time face higher medical costs, lost wages owing to illness, and treatment problems. These elements have a direct impact on patients’ QOL.

The multiple regression analyses revealed that oral treatment was a good prediction for the QOL. Insulin users experienced fewer issues and a higher QOL in a study conducted in Brazil (44). This could be owing to injection pain, a higher risk of hypoglycemic consequences, or issues with insulin delivery. It should be mentioned that the Sadeghieh study found no link between patients’ QOL and the type of medication they were taking (45). This could be owing to the varied statistical population of the study.

### Limitations

The current study had a number of limitations. First, the cross-sectional nature of the present study precluded the examination of causality. Second, the results of this study can be generalized only to similar samples and not beyond. Finally, utilizing self-reported questionnaires in surveys may lead to respondents’ underestimation or overestimation of their health-related QOL, in turn, may affect the study findings.

The current study cannot be extended to all elderly patients with diabetes because it was conducted among “elderly patients with diabetes” referred to the Imam Hospital Diabetes Clinic in Ardabil (such as patients who are receiving care at home). Because many patients with diabetes are cared for in other locations such as rural areas, the study’s lack of access to their individual and socioeconomic characteristics was another limitation. Given that the present study only looks at the QOL of elderly patients with diabetes, it is advised that future studies look at the QOL of other patients with diabetes, depending on problem areas in diabetes.

## Conclusion

Individual characteristics and factors connected to health services have the largest impact on the QOL, according to the findings of this study; thus, it is expected that treatments related to these factors will improve the QOL of the elderly with type 2 diabetes. Our recommendation is that, in addition to regular physician appointments, these patients’ unpleasant feelings associated to diabetes be evaluated.

### Implications

The most effective clinical method in evaluating the QOL problems of diabetic elderly people is to pay attention to the psychological anguish produced by diabetes and to identify it quickly. The outcomes of this study will aid officials in developing and implementing scientifically sound policies to improve the QOL of “elderly peaple with diabetes.” Psychological distress and treatment barriers must be highlighted in interventions.

### Strengths of study

This study supports the validity, reliability, and responsiveness of the problem areas in PAID and the SF-12 in modeling health outcomes for health practitioners and the health institution management of type 2 diabetics.

## Data availability statement

The raw data supporting the conclusions of this article will be made available by the authors, without undue reservation.

## Ethics statement

The studies involving human participants were reviewed and approved by Ardabil University of Medical Sciences. The patients/participants provided their written informed consent to participate in this study. Written informed consent was obtained from the individual(s) for the publication of any potentially identifiable images or data included in this article.

## Author contributions

HM: The concept of study/design, helping to collect data, analysis, and preparig a manuscript. ES with a detailed review of the proposal and article design. A-HS Study design, important reviews for important intellectual content, data analysis, monitoring, and final review.

## Conflict of interest

The authors declare that the research was conducted in the absence of any commercial or financial relationships that could be construed as a potential conflict of interest.

The reviewer HO declared a shared affiliation with the author(s) ES, AS to the handling editor at the time of review. The reviewer EM declared a shared affiliation with the author(s) HM to the handling editor at the time of review.

## Publisher’s note

All claims expressed in this article are solely those of the authors and do not necessarily represent those of their affiliated organizations, or those of the publisher, the editors and the reviewers. Any product that may be evaluated in this article, or claim that may be made by its manufacturer, is not guaranteed or endorsed by the publisher.
